# Supplementary figures and images for: Genomic analysis of Shiga toxin-producing Escherichia coli from patients and asymptomatic food handlers in Japan
Source: PLoS One. 2019 Nov 19;14(11):e0225340. doi: 10.1371/journal.pone.0225340 (PMC6863542; doi:10.1371/journal.pone.0225340)

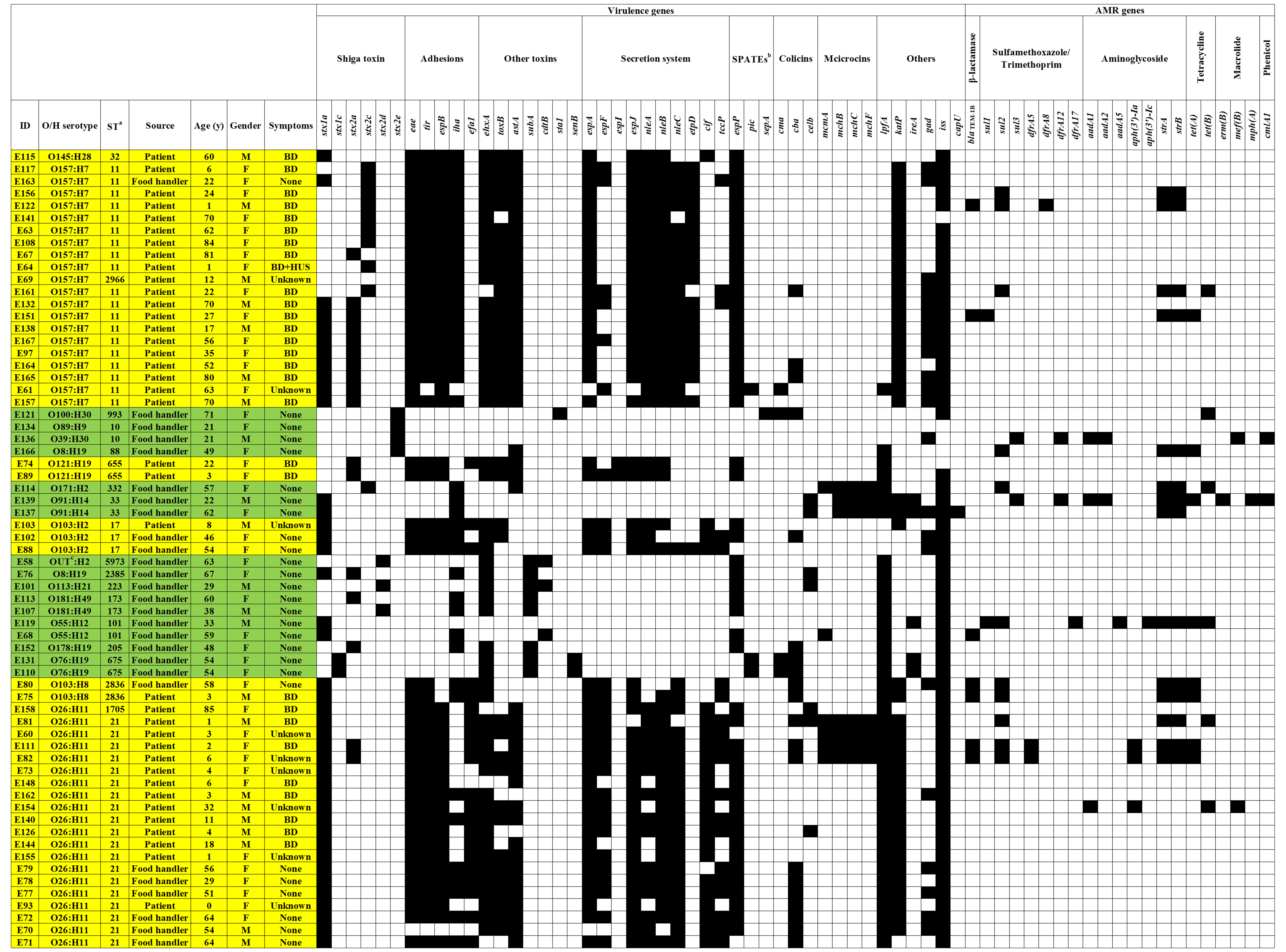

Supplement: S1 Fig — Yellow shading = isolates with major serotypes; light green shading = isolates with minor serotypes. The presence (black) or absence (white) of virulence genes and AMR genes is shown. aST: sequence type. bSPATE: Serine protease autotransporters of Enterobacteriaceae. cOUT: O-serotype untypable. (TIF) [file pone.0225340.s001.tif]
