# Supplementary material for: Genomic analysis of Shiga toxin-producing Escherichia coli from patients and asymptomatic food handlers in Japan
Source: PLoS One. 2019 Nov 19;14(11):e0225340. doi: 10.1371/journal.pone.0225340 (PMC6863542; doi:10.1371/journal.pone.0225340)
Supplement: Attachment — Submitted filename: Response to Reviewers.docx [file pone.0225340.s005.docx]

**Department of Infectious Diseases**

**Tohoku University Graduate School of Medicine**

1-1 Seiryo-machi, Aoba-ku,

Sendai, Miyagi Prefecture 980-8574, Japan.

Dr. Joerg Heber

Editor-in-Chief of PLOS ONE

Dr. Pina Fratamico

Academic Editor of PLOS ONE

Re: Manuscript ID: PONE-D-19-20606R1

Dear Dr. Heber and Dr. Fratamico,

We thank you very much for your advice on resubmitted version of our manuscript entitled **“Genomic analysis of Shiga toxin-producing *Escherichia coli* from patients and asymptomatic food handlers in Japan”** (PONE-D-19-20606R1) and inviting a revision. We are pleased that you are grateful for your thoughtful comments.

We have addressed each of the editor’s concerns in a point-by-point response, with each comment and response numbered sequentially as C and R. Each comment is stated in bold font, and our response is in standard font.

We again thank the editors and the reviewers for their careful review of our manuscript and hope that the manuscript is now acceptable for publication in PLOS ONE.

Hiroaki Baba, M.D., PhD

Department of Infectious Diseases, Tohoku University Graduate School of Medicine

1-1 Seiryo-machi, Aoba-ku, Sendai, Miyagi Prefecture 980-8574, Japan

TEL: 81-22-717-7373

E-mail: hbaba48@med.tohoku.ac.jp

**Our responses to the comments from the Editors:**

**The revised manuscript is improved; however, additional changes that are needed are the following:**

**C-1**

**Line 20: comma after disease.**

**Line 29: comma after tir.**

**Line 30: comma after nleB.**

**Line 44: change causes to cause.**

**Line 46: change to - …..serotype, severe infections caused by non-O157 serogroups are…..**

**Line 64: change to ….pathogenicity and antibiotic resistance….**

**Line 118: change to ….. (AE005174), STEC O157:H7…..**

**Line 122: change to ….A phylogenetic tree….**

**Line 124: change to ……and it was visualized…..**

**Lines 137-141: change to …….STEC strains. An O-group was not detected in the one OUT (O-serogroup untypable) isolate by SerotypeFinder. In this isolate, no O-processing genes (wzx, wzy, wzm, wzt) were detected by BLAST. It is possible that this isolate could be assigned to serogroups O14 or O57 since O-processing genes for these serogroups have not been found in their genomes [20], or it could represent a new serogroup.**

**Line 323: change to ……to provide a better……..**

**Line 347: Int J Infect Dis.**

**Line 351: italicize gene name.**

**References #13, 20, 25, 35, 38 – The first letter of the words in the title of the paper should not be capitalized.**

**References #20 and 35 – italicize Escherichia coli.**

**Line 417: italicize stx**

**Line 418: Front Cell Infect Microbiol.**

**Line 435: Euro Surveill.**

R-1 We thank the editor for pointing out grammatical and typographical errors and have corrected these throughout the manuscript.

**C-2 Line 157: In Figure 1, O145:H28 is grouped as a minor serotype, not a major serotype. Please clarify/correct.**

R-2 We thank the editor for pointing out this error. We grouped O145:H28 as a major serotype in Figure 1 (Please see revised Figure 1).

**C-3 Lines 194-197: This sentence is still not clear as written. To what do the figures 41/48 and 47/48 refer (which genes or groups of genes)? Please rewrite this sentence based on what is shown in Table 2.**

R-3 We thank the editor for this comment. We have changed this sentence to “Among the 48 isolates with major serotypes, adhesion genes *eae*, *tir*, and *espB* were detected in 47 (98%), 46 (96%), and 45 (94%) isolates respectively, and secretion system genes *espA*, *espJ*, *nleA*, *nleB*, and *nleC* were detected in 46 (96%), 46 (96%), 41 (85%), 45 (94%), and 41 (85%) isolates respectively” (p.14, lines 197-200).

**C-4 Line 394: Is this written correctly?**

R-4 We thank the editor for this comment. We have changed reference #19 to “Stamatakis A. Using RAxML to infer phylogenies. Curr Protoc Bioinformatics. 2015;51: 6.14.1-14.
